# Comparative Genome Sequencing Analysis of Some Novel Feline Infectious Peritonitis Viruses Isolated from Some Feral Cats in Long Island

**DOI:** 10.3390/v17020209

**Published:** 2025-01-31

**Authors:** Abid Ullah Shah, Blanca Esparza, Oscar Illanes, Maged Gomaa Hemida

**Affiliations:** Department of Veterinary Biomedical Sciences, College of Veterinary Medicine, Long Island University, Brookville, NY 11548, USA; abidullah.shah@liu.edu (A.U.S.); blanca.esparza@liu.edu (B.E.); oscar.illanes@liu.edu (O.I.)

**Keywords:** feline coronavirus, FCoV, feline infectious peritonitis, FIPV, histopathology, IHC, isolation, qRT-PCR, IFA, next generation sequencing (NGS), phylogenetic analysis

## Abstract

Feline infectious peritonitis virus (FIPV) remains as one of the leading causes of morbidity and mortality in young cats from shelters and catteries worldwide. Since little is known about the molecular characteristics of currently circulating FIPV strains in Long Island, New York, samples from two shelter cats submitted to the Pathology Diagnostic Services of the Long Island University College of Veterinary Medicine, with gross and microscopic lesions consistent with those of FIP were processed for virus isolation, molecular characterization and full-length genome decoding. The younger shelter cat, a 1-year-old male (A15) was found dead without previous signs of illness. Postmortem examination revealed gross and microscopic lesions characterized by vasculitis, necrosis, hemorrhage, and pyogranulomatous inflammation confined to the colon and associated lymph nodes. The second cat, a 7-year-old spayed female (A37) had an identical clinical history and similar but widespread lesions, including fibrinous peritoneal effusion, cecal, colonic, renal, and hepatic involvement. The gross and microscopic diagnosis of FIP in these cats was confirmed by immunohistochemistry (IHC) demonstration of feline coronavirus antigen using mouse anti-FIPV3-70 monoclonal antibody. Virus isolation from saved frozen kidney and colon tissue was performed through several subsequent blind passages in MDCK and Vero cell lines. Confirmation of the FIPV isolation was done through qRT-PCR, IFA, western blot using N protein antibodies, and NGS of the full-length genome sequencing. The full-length genome sequences of the virus isolate from the two cats were decoded using next-generation sequencing (NGS) and deposited in the GenBank as accession numbers PQ192636 and PQ202302. The genome size of these isolates was (29355 and 29321) nucleotides (nt) in length, respectively. While their genome organization was consistent with other FIPV genomes as follows (5’UTR-ORF1ab-S-3abc-M-E-7b-3’UTR-3’), marked differential mutations were observed in the ORF1a/b, S, 3Abc, and 7b protein genes of the two FIPV isolates. One notable deletion of 34 nucleotides was observed in the 7b genes of one of these isolates but was absent in the other. We confirmed the potential recombination events during the evolution of those two FIPV field isolates with the potential parent virus as FECoV-US isolated in 1970 and the potential minor parent as the Canine coronavirus. Our results provide a comprehensive molecular analysis of two novel FIPV isolates causing fatal disease in shelter cats from Long Island. Diagnostic surveillance with molecular characterization and sequencing analysis of circulating FIPV strains within animal shelters may help early detect unique emerging clinical and pathological manifestations of the disease and develop more targeted prophylactic and therapeutic approaches to control it.

## 1. Introduction

The feline coronavirus (FCoV) belongs to the order *Nidovirales*, family *coronaviridae*, and genus Alphacoronavirus-1 [1]. The viral genome consists of a single molecule of single-strand RNA ranging from 29 to 30 kilobases in size. The viral genome has a typical coronavirus genome, including two untranslated regions flanking the entire genome at the 5′ and 3′ end, respectively. The ORF1ab occupies the 5′ two-thirds of the genome with a ribosomal frameshifting in between. The viral protease further processes this long ORF1ab into several non-structural proteins (NSP-1-NSP16). The major structural proteins reside at the 3′ end of the genome and are interspersed by some non-structural proteins. There are five non-structural proteins (3a–3c, 7a, and 7b). The FCoV is classified into two major serotypes (I and II) based on their spike glycoprotein sequences and serum neutralization assays [2]. The pathogenesis and emergence of the FIPV in cats is complex and still not yet well established. The currently accepted theory is that about 10 % of cats infected with FCoV may develop FIPV clinical diseases [3] when the virus is subjected to several mutations across the viral genome in the body of infected cats, particularly in some key regions of the spike glycoprotein and some other accessory proteins such as the 3C and 7b nonstructural proteins [4,5].

There are three possible outcomes of FCoV infection in cats. (1) Some cats can shed the virus for a very short time or never shed the virus. (2) Some cats may shed the virus in their body secretions and excretions for 2–3 months, stop shedding it, or intermittently shed it (70–80% of the affected cat population). (3) Some cats (10–15%) become long-term shedders [6]. FIPV mainly infects young cats less than two years old (55–67%), and susceptibility to infection decreases progressively with age [7]. Kittens in highly dense populations are most likely to get infected with FCoV during their first two weeks, primarily by the fecal–oral route [2]. A more recent study reported cross-species transmission of the serotype-1 FCoV between the wild and domestic cat populations in the USA [8]. The main tissue tropism of FCoV is towards the cells of the enteric mucosa, infecting cells starting from the lower sections of the small intestine to the cecum [9,10]. This contrasts with FIPV, which mainly targets monocytes and macrophages, which are cells that replicate the virus and play an essential role in viral molecular pathogenesis and lesion development [11]. With the exception of monocytes, leucocytes do not seem susceptible to FIPV infection. However, they have a pivotal role in the pathogenesis of endothelial damage in small veins, leading to phlebitis and peri-phlebitis, the hallmark microscopic lesions of FIPV infection [6,12].

FCoV infection usually flourishes in a crowded cat population, particularly in shelters. Although FCoV and FIPV are closely related in their genomic composition despite some reported mutations and recombination, they manifest in two different clinical syndromes in the affected cats. The FCoV serotype-I is the most prevalent among the cat population. Only 7–14% of the FCoV-infected cats would develop the FIPV syndrome [2]. Some studies showed that 20–60% of the tested domestic cats seroconverted to FIPV and that in shelter cats, the seroconversion rate can be as high as 90% [10]. There are three possible outcomes of FCoV infection in cats. (1) Some cats can shed the virus for a very short time or never shed the virus. (2) Some cats may shed the virus in their body secretions and excretions for 2–3 months, stop shedding it, or intermittently shed it (70–80% of the affected cat population). (3) Some cats (10–15%) become long-term shedders [6]. FIPV mainly infects young cats less than two years old (55–67%), and susceptibility to infection decreases progressively with age [7]. Kittens in highly dense populations are most likely to get infected with FCoV during their first two weeks, primarily by the fecal–oral route [2]. FCoV infection is often subclinical or associated with a mild transient intestinal infection. In contrast, FIPV is much more lethal due to the often-severe vasculitis leading to body cavity effusions and widespread fibrinous and pyogranulomatous serosal and parenchymal lesions in multiple organs [13]. However, the definitive differential diagnosis of FIP from FCoV infection is challenging under field conditions since it requires careful assessment of the suspected cats’ history, clinical signs, and hematological and biochemistry profiles [6,12]. This situation makes FIPV disease surveillance, particularly serosurveillance studies, very difficult. One FIPV study in the USA in 2013 compared the viral genome sequence of one isolate after several blind passages in cell culture [14]. This study showed that subsequent cell culture passages resulted in mutations in the viral genome, particularly involving the S, 3A, 3C, 7a, and 7b genes [14]. Little is known about the actual prevalence of FIPV in the USA’s feline population or the possible clinical implications associated with spontaneous mutations of the virus. The main objectives of this study were to perform isolation and comprehensive characterization of some field isolates of FIPV currently circulating in the Long Island shelter-cat population, decode their full-length genome sequencing, and compare it with the USA and worldwide FIPV and FCoV sequences available in the GenBank.

## 2. Materials and Methods

### 2.1. Sample Collection from Some Suspected FIPV-Infected Feral Cats

During the period from January to April 2024, frozen tissue specimens from two deceased shelter feral cats (A15 and A37), necropsied at the Long Island University College of Veterinary Medicine and with gross and microscopic lesions highly suggestive of FIP, were processed for virus isolation. Frozen liver, kidney, and colon samples from each animal were divided into two: one was kept at (−80 °C) for further molecular testing, and the other was kept in 10% buffered formalin for immunohistochemistry (IHC) examination, as described below. The FIPV reference strain (79-1146, accession No. AY994055) was obtained from BEI Resources (National Institute of Allergy and Infectious Diseases (NIH/NIAID) supported program managed by ATCC, Cat. No. NR-43287) and was used as a positive control throughout the study.

### 2.2. Processing of the Collected Tissue Specimens for Histopathology and IHC Examination

At necropsy, selected tissue samples were preserved in 10% buffered formalin, later trimmed, and stained with hematoxylin and eosin (HE), as previously described [15]. Deparaffinized tissue sections were processed for IHC using the CoV-N protein antibodies FCoV-nucleocapsid (FCoV-N) and mouse anti-FCoV monoclonal antibody (Invitrogen, Waltham, MA, USA; Clone: FIPV3-70, Cat. # MA1-82189), as previously described [16]. Both the H&E-stained sections and the IHC slides were examined by light microscopy.

### 2.3. Processing of the Collected Feline Specimens for Molecular Analysis

Tissue specimens from each cat were pooled as one sample, and a 10% tissue suspension was prepared from each tissue pool. Briefly, one gram of the pooled tissue specimens was finely chopped and homogenized using 1.5 mm beads (Merck, Kenilworth, New Jersey, USA; Lot. No. 3110) and PBS containing 1% penicillin/streptomycin cocktail (Gibco, Brooklyn, NY, USA; Lot. No. 2321161) with BeadBug6 microtube homogenizer (Benchmark, New York, NY, USA). The supernatants were centrifuged and passed through 0.45 um filters (Cytiva, Marlborough, MA, USA; Lot. No. 18008685). The purified filtered tissue suspensions were stored at (−80 °C) for further analysis.

### 2.4. In Vitro Propagation and Isolation of the New FIPV Field Isolates Using Cell Lines

Each purified filtered sample was incubated independently with the Thermo Scientific Pierce TPCK Trypsin (Thermo Fisher Scientific, Waltham, MA, USA; Ref. No. 20233), diluted in plain DMEM medium, and then incubated at (37 °C) for 30 min with gentle rocking every 10 min. Three cell lines were used to isolate and propagate these two new FIPV field isolates. The Madine–Darby Canine Kidney (MDCK) and the Vero cells were inoculated with the purified samples, prepared as above, from the organs of each cat for 1 h at 37 °C and 5% CO_2_. The infected cells were incubated with a complete medium. The complete medium for MDCK and Vero cells is composed of EMEM (ATCC; 30-2003), supplemented with 10% Fetal Bovine Serum (FBS) (Gibco; Lot. No. 2565838RP) and 1% streptomycin and penicillin antibiotics. Infected cells were observed daily under the inverted microscope for five days or until cytopathic effects (CPE) were observed. The FIPV-79-1146 reference strain was used to control the propagation and isolation of the FIPV field isolates using MDCK and Vero cell lines.

### 2.5. Isolation of the FIP-RNAs and the Confirmation of the Viral Identity by the Quantitative Reverse Transcriptase Polymerase Chain Reaction (qRT-PCR)

The viral RNAs were extracted from the processed tissue specimens, as previously described [17]. The total RNAs were isolated using TRIzol LS Reagent (Invitrogen; Ref # 10296010), as per the kit’s instructions. The extracted viral RNAs were converted into complementary DNA (cDNA) using the high-capacity reverse transcription kit (Applied Biosystems, Foster City, CA, USA, Lot. No. 2902953s). The cDNA was used to perform the qRT-PCR using the PowerUp™ SYBR™ Green Master Mix (Applied Biosystems, Lot. No. 2843446), as per the kit’s instructions. The FIPV-specific oligonucleotides are (FIPV-forward-5′-AGCAACTACTGCCACRGGAT-3’ and FIPV-reverse 5′-GGAAGGTTCATCTCCCCAGT-3′) [18]. The primers for the B-actin (Forwared-5′-ACTGGGACGACATGGAGAAG-3′ and reverse 5′-TGGGGTGTTGAAAGTCTCGA-3′) were used as a positive control. The primers used in this study were designed using the Primer3 online software (https://primer3.org/) [19]. The qRT-PCR was performed using the Quant Studio3 System (Applied Biosystems). As previously described, the FIPV genome expression levels were normalized to the β-actin using the 2^−ΔΔCt^ method [20].

### 2.6. Detection of the FIPV Antigens by the Fluorescence Immunoassay

The IFA was conducted on the FIPV-infected cat tissues and the cell lines infected with the FIPV field isolates (A15 and A37). All the tested tissue specimens and the FIPV-infected MDCK with the FIPV reference strain or the FIPV 79-1146, A15, and A37 were subjected to IFA analysis, as previously described [21]. The prepared tissues or the FIPV-infected cell lines were incubated with primary anti-FCoV-nucleocapsid mouse anti-feline monoclonal antibody (Invitrogen, Clone: FIPV3-70; Cat. # MA1-82189) overnight in dark chambers at 4 °C. The prepared tissues or the FIPV-infected cell lines were incubated in dark champers for 1 h with the secondary antibody goat anti-mouse conjugated with Alexa-Flour 488 (Invitrogen, Clone: gG2a (y2a), Ref. # A21131) [21]. DAPI (Invitrogen, Ref. # D1306) was used as a counterstain, which was incubated for 5 min with each slide. As previously described, the prepared sections were examined using the fluorescent microscope (ZEISS LSM 900, Oberkochen, Germany) [21].

### 2.7. Detection of the FIPV Proteins Using the SDS-PAGFE and the Western Blot Assay

The infected MDCK (mock or FIPV) cells were lysed, as previously described [21]. The extracted total protein concentrations were measured using the Pierce BCA protein assay kit (Thermo Scientific, Lot. No. YD366108), as per the kit’s instructions. The proteins were quantified using the Nano-Drop OneC (Thermo Scientific). The protein samples were mixed with an equal volume of the 2× laemmli sample buffer (Bio-Rad, Hercules, California, USA, Cat. # 1610737) and incubated for 10 min at (100 °C). The protein samples and the iBright Pre-stained Protein Ladder (Invitrogen, Lot. # 2666356) were loaded onto the SDS-PAGE and then electrophoresed at 100 volts for 90 min. The total protein was visualized by staining the SDS-PAGE with the Brilliant Blue R (Sigma, Burlington, MA, USA, Lot. # MKCJ2486), as per the kit’s instructions. The images were captured using the Gel-Doc Go Imaging System (Bio-Rad). The molecular weight of the FIPV proteins was estimated using the molecular weight calculator (https://www.bioinformatics.org/sms/prot_mw.html) online tools.

The Western blot (WB) analysis was performed, as previously described [21]. Briefly, the protein’s blotted membranes were incubated with horseradish peroxidase (HRP)-conjugated secondary antibodies for 1 h at room temperature. Finally, the membranes were treated with the Clarity Western Enhanced Chemiluminescence (ECL) (Bio-Rad, Cat. # 170-5060) and visualized using the GelDoc Go Imaging System (Bio-Rad). The primary antibodies used in this study were the FCoV-nucleocapsid (FCoV-N) mouse anti-feline monoclonal antibody (Invitrogen; Clone: FIPV3-70, Cat. # MA1-82189) and the β-actin rabbit anti-bovine polyclonal antibody (Invitrogen; Cat. # PA1-46296). The secondary antibody used in this experiment was the HRP-conjugated IgG (H+L) goat anti-mouse (Invitrogen; Ref. # 31430).

### 2.8. Decoding of the Full-Length Genomes of the Novel Field FIPV Isolates Using the Next Generation Sequencing (NGS)

The MDCK propagated FIPV cell-culture supernatants were used for the RNA extraction, as described above (Section 2.5) [21]. Briefly, the isolated RNAs from the cell-culture-propagated FIPV field isolates were treated with DNase-I (RNase-free, New England Biolabs (NEB, Ipswich, ME, USA), Lot. # 10213692) to remove any residual host DNA contamination. The treated RNA samples were submitted to Azenta Life Sciences (South Plainfield, NJ, USA) to decode the full-length genome of these two FIPV isolates. The NGS procedure was carried out as described elsewhere [21]. Briefly, the NGS was carried out on the isolated RNAs from the new FIPV isolates (A17 and A37). The NGS was conducted in two consecutive steps. First, the RNA libraries were generated using the Ultra II RNA Library preparation kits purchased from NEB (NEBNext^®^ Ultra™ II RNA Library Prep Kit for Illumina^®^; catalog number: NEB #E7770S/L). Library preparation was carried out as per the kit’s guidelines. Second, the sequencing step was carried out using the generated libraries from the previous step. The Nova-Seq X Plus using a 2× 150 bp configuration was used to sequence the generated libraries. Third, the sequences obtained per sample were subjected to bioinformatics analysis. The Trimmomatic v.0.36 software was used to trip the ends and adaptors and remove the poor-quality sequences. The clean sequences were mapped to the reference FIPV full-length genome sequences retrieved from the GenBank (accession no. #AY994055). We used the VarScan, using a minimum coverage of 10, minimum reads of 4, minimum variant frequency of 0.5%, and a minimum *p*-value of 0.05. The assembled full-length genome sequences for each isolate were deposited in the GenBank.

### 2.9. Construction of the Phylogenetic Trees and FIPV Sequence Analysis

The obtained sequences from each FIPV isolate were assembled into a single contig and aligned to the reference strain of the FIPV. The obtained complete genomes of the new field isolates of the FIPV, their spike glycoproteins, their nucleocapsid proteins, and their non-structural protein (NSP) 7b of FIPV/FCoV, feline enteric coronavirus, canine coronavirus (CCoV), porcine respiratory coronavirus (PRCV), and transmissible gastroenteritis virus (TGEV), were used to perform the multiple sequence alignment using the Geneious Prime software Version 11.0.20.1 (https://www.geneious.com). As previously described, the MEGA11 software was used to construct the phylogenetic trees based on the full-length genomes S, N, and NSP-7b [22,23,24]. The branches of the phylogenetic trees (confidence limits) were evaluated using bootstrap (1000 replicates). The phylogenetic tree was constructed using the online software iTOL (https://itol.embl.de/) [25]. The analysis was performed on 32 selected genome sequences belonging to the Alphacoronaviruses. The GenBank accession numbers and the associated information for members of the Alphacoronaviruses that were used in the MSA are listed in Supplementary Table S1.

### 2.10. The Pairwise Analysis of FIPV Isolates from This Study with Reference Strains from the Alpha Coronavirus Group

Comparative pairwise comparison analysis of FIPV-A15 nucleotide sequences was performed with FIPV-A37 and other alpha-coronaviruses belonging to FCoV-type-I and FCoV-type-II. Multiple sequence alignment of the nucleotide sequence of the complete genome and each gene (ORF1ab, spike, 3a, 3b, 3c, envelope, membrane, nucleocapsid, 7a, and 7b) was performed using Geneious Prime v11. The heat map was constructed based on the nucleotide similarities using GraphPad Prism v9 software (http://www.graphpad.com/faq/viewfaq.cfm?faq=1362).

### 2.11. Statistical Analysis

The reported results in this study were expressed as ± standard deviation (SD). All data analyses were performed using the Mac’s GraphPad Prism version 9. The one-way analysis of variance (ANOVA) and Tukey’s test were used to compare the results and to look at the comparison between various groups. The statistically significant results were considered if the obtained results showed (*p* < 0.05). The scale of the statistical significance was assigned as follows: (** *p* < 0.01 and **** *p* < 0.0001). 

## 3. Results

### 3.1. The Gross and Microscopic Necropsy Findings in the Two FIPV-Infected Cats

**Figure 1:**
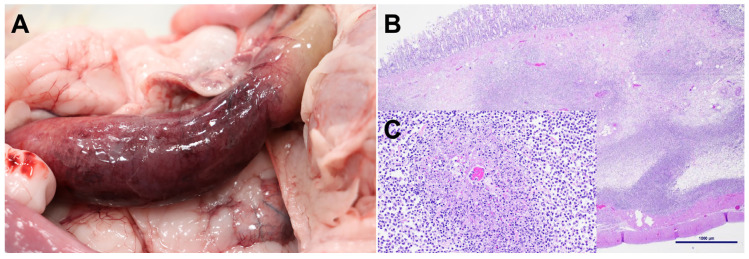
Significant necropsy findings in the FIV/A15 infected cat. (**A**) The descending colon was diffusely thickened due to transmural congestion, edema and inflammation. (**B**) Microscopically, there was marked expansion of the colonic submucosa by edema and the presence of inflammatory cells, Hematoxylin and Eosin (H&E)-stained section, scale bar: 1000 µm. (**C**) Inset: Closeup of submucosal venule surrounded by fibrin, necrotic cellular debris and inflammatory cells (fibrino-necrotizing phlebitis and peri-phlebitis). Scale bar 100µm.

**A15:** This was an approximately 1-year-old male (neutered) feral DSH cat that had been in the shelter for 2 months. There were no clinical signs of illness; this cat was found dead in his cage. Significant gross and microscopic necropsy findings were confined to the descending colon and colonic and mesenteric lymph nodes. The affected colon was diffusely dilated, with a thickened (edematous) wall and a dark red (hemorrhagic) serosal surface (Figure 1A). The affected colon contained only a small amount of pale tan pasty enteric contents and a few poorly defined areas of reddening throughout the mucosa. Colonic and mesenteric lymph nodes were mottled pale tan, moist, and moderately to markedly enlarged. Microscopically, lesions in the colon were characterized by prominent submucosal edema and multiple foci of hemorrhage and pyogranulomatous inflammatory cell infiltration, often centered around variably sized venules (Figure 1B). Fibrin deposition combined with variable amounts of cellular and nuclear debris, degenerate neutrophils, and macrophages were often present within and around the wall of venules at the center of the inflammatory foci (Figure 1C). Fibrinous and pyogranulomatous inflammation foci were present in the enlarged colonic and mesenteric lymph nodes.

**Figure 2:**
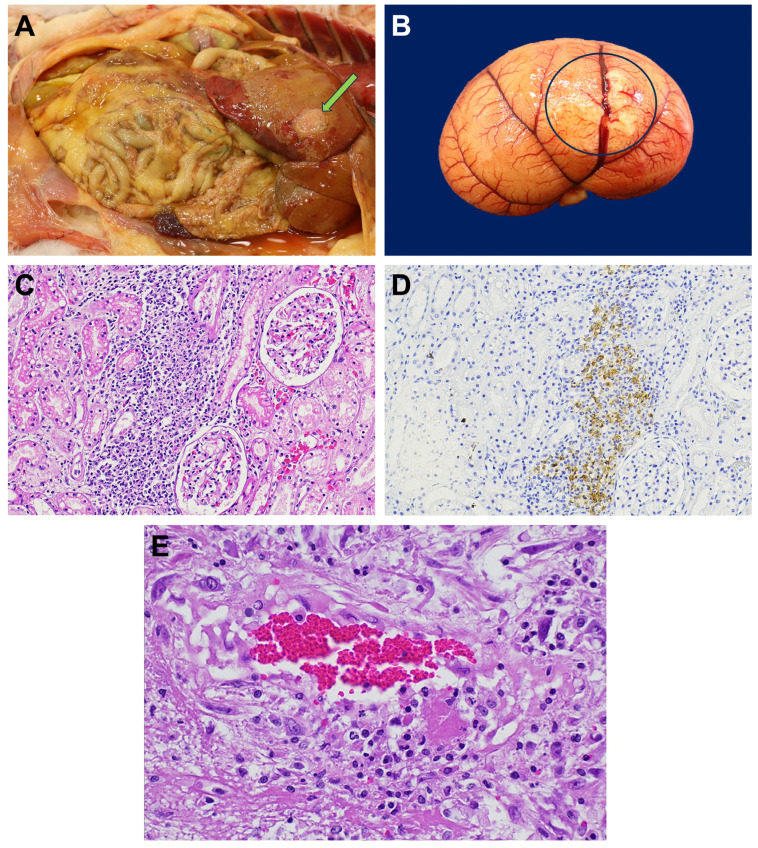
Necropsy findings in the FIPV/A37 infected cat. (**A**) In addition to peritoneal effusion, thin layers and small fibrin plaques were scattered on the liver, omentum, mesentery, and serosal surface of the intestine. Fibrin deposition on an edematous gall bladder wall resulted in soft adhesions to the adjacent liver lobes (arrow). (**B**) Slightly raised pale tan foci around blood vessels on the cortical surface of 279 the kidney (circle). (**C**) H&E-stained section of a focal area of perivascular interstitial nephritis. (**D**) IHC demonstrates positive immunostaining for FCoV antigen. (**E**) Phlebitis and peri-phlebitis, within the gall bladder wall, H&E-stained section.

**A37:** This was a seven-year-old female (spayed) DSH free-roaming cat living in a shelter. According to the shelter, she presented no signs of illness before being found dead. Significant gross necropsy findings were dehydration, icterus, and abdominal enlargement secondary to peritoneal effusion. Fibrin deposition was scattered throughout the abdominal organs, omentum, mesentery, and parietal peritoneum. The liver was diffusely pale, and the quadrate, right medial, and right lateral liver lobes were fused by the presence of fibrin, entrapping the gall bladder (Figure 2A), which appeared distended and filled with partially inspissated bile. A few small, slightly elevated, pale tan foci, which extended into the underlying parenchyma, were observed along the superficial cortical blood vessels of the kidney (Figure 2B). Microscopically, these lesions represented foci of perivascular interstitial nephritis (Figure 2C) with positive immunostaining for FeCoV antigen (Figure 2D). The ileocecal valve and the wall of the adjacent cecum and ileum were mildly thickened, and the neighboring mesenteric lymph nodes were moist and moderately enlarged. Microscopically, foci of fibrinous and pyogranulomatous inflammation with occasional phlebitis and peri-phlebitis (Figure 2E) were present in the affected abdominal organs, gall bladder, omentum, mesentery, and mesenteric lymph nodes. Necrotizing and inflammatory lesions were particularly severe in the wall of the gallbladder and the neighboring hepatic parenchyma.

### 3.2. Conformation of FIPV Infection in (A15 and A37) Tissue Specimens by IFA

**Figure 3:**
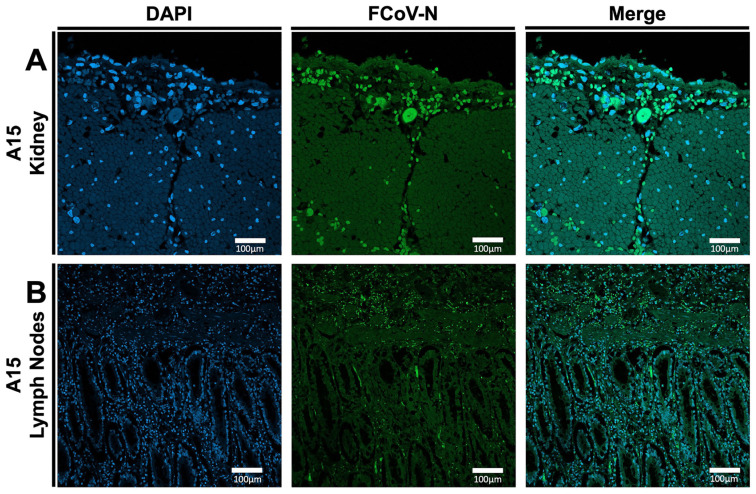
Results of the IFA on some processed tissues collected from the A15 feral cat. (**A**) IFA of the kidney samples collected from A15 FIPV-infected cat. (**B**) IFA of the lymph nodes collected from A15 FIPV-infected cat. The blue color indicates DAPI staining the cell nucleus. The green color indicates the FCoV-N protein. All the images were captured at 10× magnification. The scale represents 100 µm.

**Figure 4:**
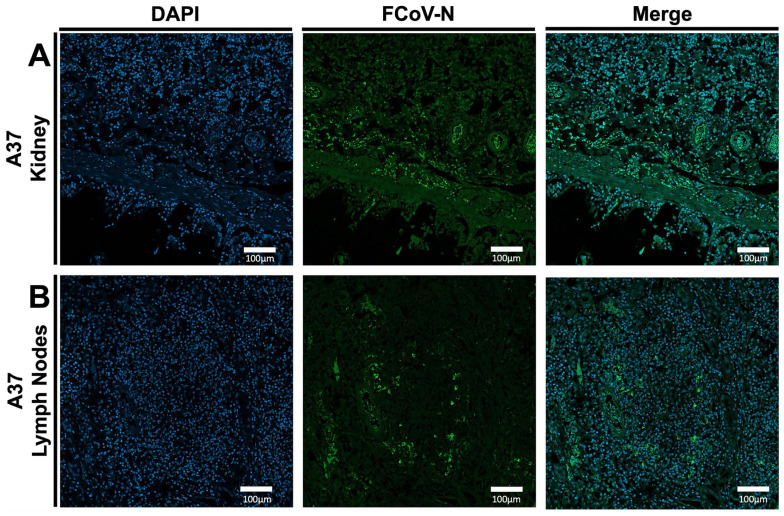
Results of the IFA on some processed tissues collected from the A37 feral cat. (**A**) IFA of the kidney samples collected from A37 FIPV-infected cat. (**B**) IFA of the lymph nodes collected from A37 FIPV-infected cat. The blue color indicates DAPI staining the cell nucleus. The green color indicates the FCoV-N protein. All the images were captured at 10× magnification. The scale represents 100 µm.

The FIPV infection in tissues collected from A15 and A37 was assessed by IFA assay using the FCoV-N fluorescent conjugated antibody. Our results showed intense green-fluorescent staining, indicating high levels of FIPV-N protein expression in both the kidney and lymph nodes of case A15 (Figure 3A,B). In the A37 kidney sample, immunofluorescence was mainly confined to the cortex of the kidneys (Figure 4A). Still, it was quite prominent in both the cortex and medulla of the sampled lymph nodes (Figure 4B).

### 3.3. Molecular Confirmation of FIPV Infection in the Cat Tissue Specimens by qRT-PCR

**Figure 5:**
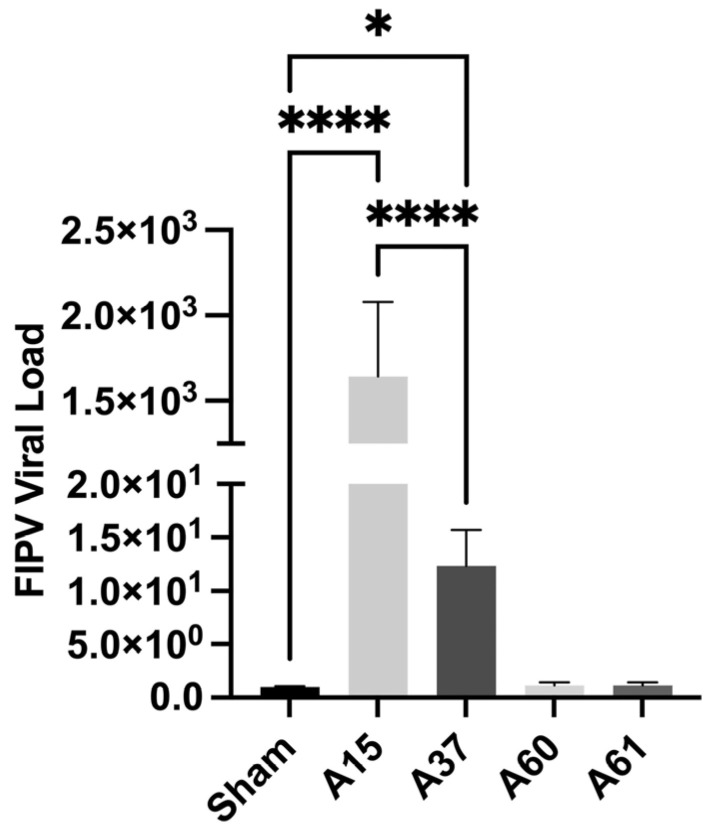
Confirmation of the FIPV infection in the tissues collected from infected feral cats: the qRT-PCT was performed on tissue samples collected from two infected feral cats (A15 and A37) for the presence of FIPV. Tissue samples collected from feral cats negative for FIPV were used as sham (control) to normalize the FIPV genome expression. *p < 0.05, **** *p* < 0.0001.

The qRT-PCR data revealed the detection of the FIPV-RNAs in the tissues of both cats (A15 and A37). The genomic FIPV load in the case of A15 was approximately 165.2 times higher than in A37 (Figure 5).

### 3.4. Virus Isolation and Propagation of the FIPV Isolates Detected in Cats A15 and A37

**Figure 6:**
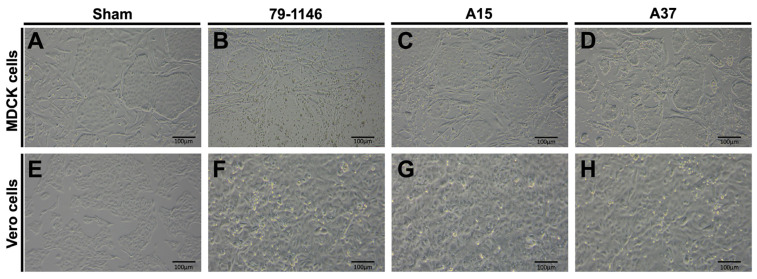
Morphological changes in the MDCK and Vero cell lines infected with the FIPV field isolates (A15 and A37). (**A**–**D**) Morphological changes in the MDCK infected with the sham, FIPV-79-1146, FIPV-A15, and FIPV-A37. (**E**–**H**) Morphological changes in the Vero cells infected with the sham, FIPV-79-1146, FIPV-A15, and FIPV-A37.

The reference strains FIPV-79-1146, FIPV-A15, and FIPV-A37 were cultured in MDCK and Vero cell lines for three subsequent passages. Our results showed that the FIPV 79-1146 induced the production of the cytopathic effect (CPE) in the form of cell detachment from the monolayer and cell rounding and swelling at 72 h post-infection (hpi) when compared to the sham infected MDCK cells (Figure 6A,B). Similarly, the field isolates (FIPV/A15 and FIPV/A37) showed a strong CPE in the MDCK at 72 hpi (Figure 6C,D). The FIPV 79-1146, FIPV/A15, and FIPV/A37 also showed CPE in the Vero cells at 72 hpi (Figure 6E–H). However, the CPE in cells infected with the reference strain of FIPV 79-1146 was more prominent and developed earlier than the CPE induced by the new FIPV isolates (A15 and A37), especially in the MDCK cells (Figure 6B–D).

### 3.5. Confirmation of the Replication and Propagation of the Field Isolates of FIPV (A15 and A37) by Immunofluorescence Assay (IFA)

**Figure 7:**
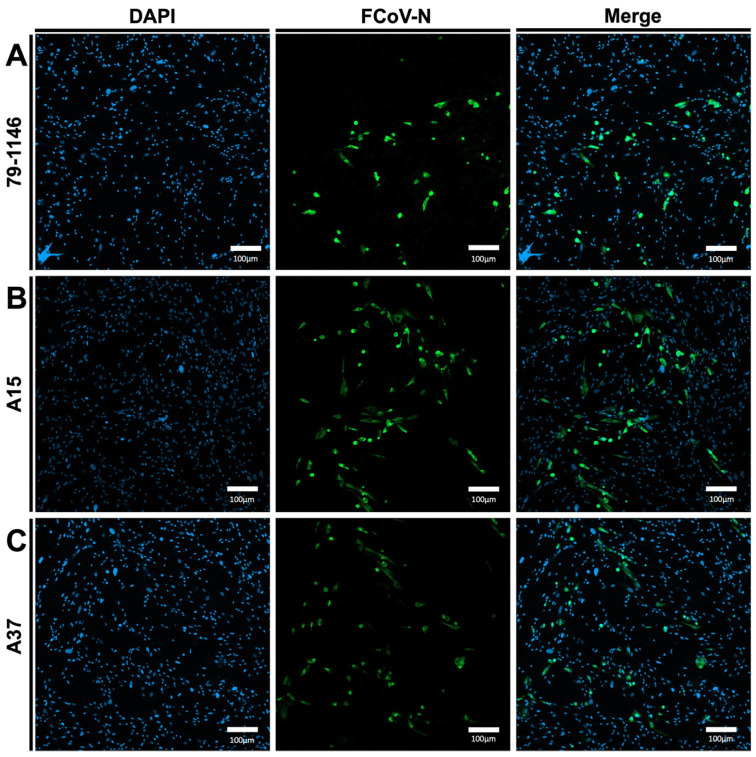
Conformation of FIPV infectivity in MDCK cells through immunofluorescence analysis (IFA): (**A**) the IFA of the MDCK cells infected with FIPV reference strain 79-1146; (**B**) FIPV-A15; and (**C**) FIPV-A37. The blue color indicates DAPI staining the cell nucleus. The green color identifies the FCoV-N. The MDCK cells were infected with the specific virus at (MOI = 1), and IFA was performed after 72 hpi.

Our results show the expression of FIPV/N proteins in the MDBK cells infected with either the FIPV/A15 or the FIPV/A37 field isolates, as demonstrated by the green, fluorescent color (Figure 7A–C). Cells infected with the FIPV/A15 isolate showed marked expression of the N protein compared to the FIPV/A37 and the positive control (FIPV-79-1146).

### 3.6. Titration of the FIPV Genome Copy Numbers in Subsequent Passages of the A15 and A37 Field Isolates Through Various Cell Lines by qRT-PCR

**Figure 8:**
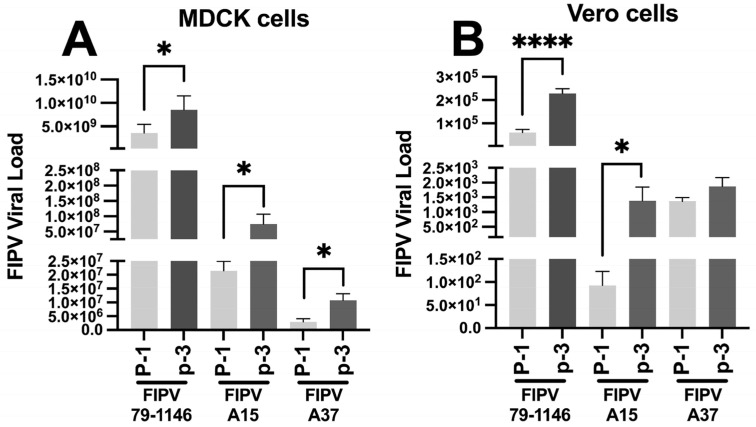
Monitoring of the propagation and isolation of the FIPV/A15 and FIPV/A37 field isolates in the MDCK and Vero cell lines by the qRT-PCR: (**A**) the genome copy numbers of the MDBK cells infected with either the FIPV/A15 or FIPV/A37 field isolates in the MDCK (P-1–P-3) compared to the sham and the FIPV-79-1146 by the qRT-PCR. (**B**) Results of the genome copy numbers of Vero cells infected with either the FIPV/A15 or FIPV/A37 field isolates in the Vero (P-1–P-3) compared to the sham and the FIPV-79-1146 by the qRT-PCR. *p < 0.05, **** *p* < 0.0001.

The qRT-PCR was performed on the MDCK and Vero cell lines infected with the positive control (FIPV 79-1146), FIPV A15, and FIPV A37 at viral passage 1 (P-1) and passage 3 (P-3). The results showed that the genomic viral load of all three samples was much higher in the MDCK cells than in the Vero cells (Figure 8A,B). Among the infected groups, FIPV 79-1146 exhibited a higher viral load than A15 and A37 in both cell lines. In the MDCK cells, the viral load of FIPV 79-1146 at P-3 was 2.41 times higher than at P-1 (Figure 8A). The FIPV A15 genomic viral load increased by about 3.52 times in the third passage (P-3) compared to P-1 (Figure 8A). Similarly, in the case of the A37 FIPV-infected MDCK cells, the viral load increased by approximately 3.57 times from P-1 to passage 3 (Figure 8A). In the Vero cells, the genomic viral load of FIPV 79-1146 increased by about 3.77 times in P-3 compared to passage 1 (Figure 8B). In the case of the A15 FIPV-infected Vero cells, the genome copy numbers increased by about 15 times in P-3 compared to passage 1 (Figure 8B). However, in the case of FIPV-A37, only a mild increase in the genome copy number was observed in Vero-infected cells in P-3 compared to passage 1 (Figure 8B). Overall, the FIPV genome copy numbers in the MDCK cells were higher for A15 than A37, whereas in the Vero cells, the FIPV A37 isolates exhibited higher genome copy numbers than in the A15.

### 3.7. Identification of the Major Structural Proteins of the FIPV-A15 and FIPV-A37 Field Isolates Using the SDS-PAGE and Western Blot Assay

**Figure 9:**
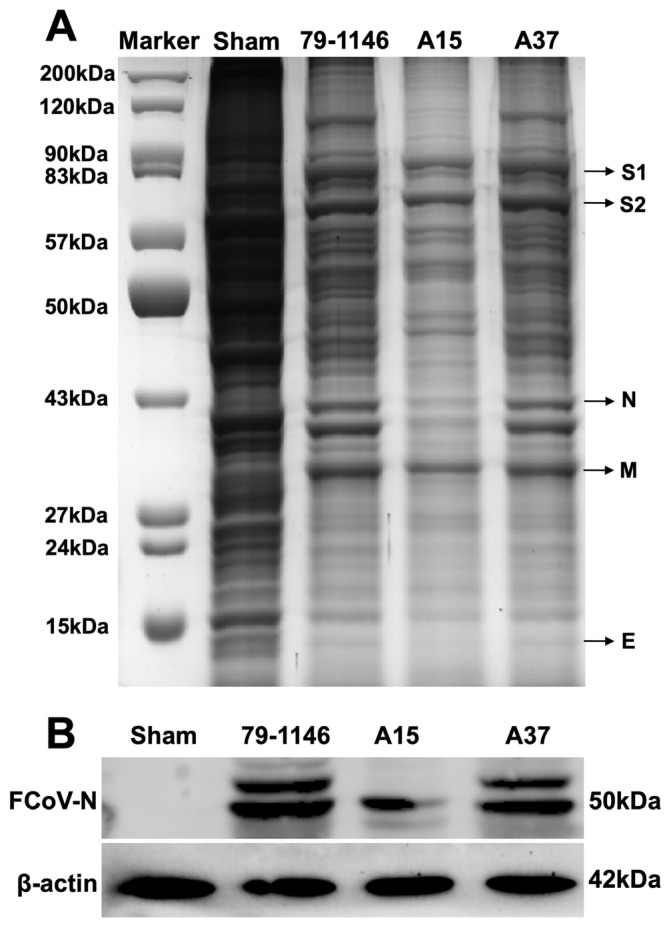
Conformation of expression of the FIPV proteins by the SDS-PAGE and WB analysis: (**A**) the SDS-PAGE results showing the total proteins in the case of the mock-infected cells: the FIPV 79-1146, the FIPV-A15, and the FIPV-A37 infected MDCK cells. Lane-1 is the ladder; lane-2 represents the mock-infected MDCK cells; lane-3 is MDCK cells infected with FIPV 79-1146; lane-4 is MDCK cells infected with FIPV-A15; and lane-5 represents the total proteins of the MDCK cells infected with FIPV-A37. (**B**) Western blot analysis of FCoV-nucleocapsid (FCoV-N) protein and β-actin protein expression in MDCK cells infected with sham, FIPV 79-1146, FIPV-A15, and FIPV-A37. MDCK cells were infected with the specific virus at (MOI = 1) for 72 hpi, and the cells were lysed and used for protein deduction.

The SDS-PAGE results showed the calculated sizes of the FIPV major proteins (spike glycoprotein-subunit 1 (S1) was ~82.8 kDa). The S2 protein was ~74 kDa, the nucleocapsid (N) protein was ~42.7 kDa, the membrane (M) protein was~29.8 kDa, and the envelope (E) protein was ~9.5 kDa (Figure 9A). The A15 infected cells showed a weak band of N protein compared to FIPV 79-1146 and A37 (Figure 9A). The Western blot analysis further confirmed the FIPV-N protein expression using a specific antibody for the FCoV-N protein (Figure 9B). Like the SDS-PAGE, the Western blot results also showed the expression of the N protein in the A15 and the A37 FIPV-infected groups of cells compared to the reference strain (Figure 9B).

### 3.8. Establishing the Genome Structure and Organization of the New Field Isolates (FIPV-A15 and FIPV-A37)

**Figure 10:**
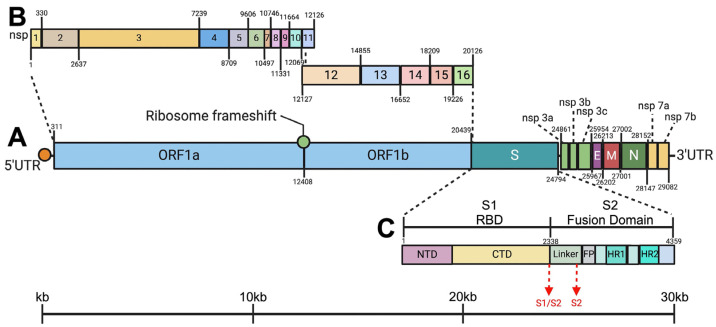
A schematic representation of the structure and organization of the newly isolated FIPV-A15 and FIPV-A37 field isolates. (**A**) The proposed model for the genome structure and organization of the FIPV-A15 and FIPV-A37 isolates using the data of the complete genome sequences. The genome is organized as follows: the 5′UTR; the Gene-1; the spike (S); the non-structural proteins (NSPs) 3a, 3b, and 3c; the envelope (E); the membrane (M); the nucleocapsid (N); the NSP 7a; the SP7b; and the 3′UTR. (**B**) A model of the organization of the 16 NSPs of the FIPV isolates was reported in this study. (**C**) A diagram showing the proposed structure of the spike glycoprotein (S) highlighting its relative subunits, sizes, and positions. The S1 subunit and S2 subunit of the spike gene, with the S1/S2 cleavage site, are in between. The S2 cleavage site is shown in red. The S1 subunit or Receptor Binding Domain (RBD) contains an N-terminal domain (NTD) and a C-terminal domain (CTD). The S2 subunit or Fusion Domain contains Linker, Fusion Peptide (FP), Heptad Repeat 1 (HR1), and Heptad Repeat 2 (HR2).

The complete genome sequences of FIPV isolates A15 and A37 were drafted using the Next Generation Sequencing (NGS). The reported sequences were deposited in the GenBank (accession numbers: PQ192636 and PQ202302, respectively). The full-length genome of the FIPV-A15 isolate was 29,355 nucleotides in length and had a GC content of 38.1%. The FIPV-A37 full-length genome was 29,320 nucleotides with a GC content of 38.1%. The Gene-1 predominantly resides at the 5′ region of the genome and comprises two large open reading frames (ORFs), ORF1a and ORF1b, flanked with a ribosomal frameshift in between (Figure 10A). This ORF1a/b encodes 16 non-structural proteins (NSPs) (Figure 10B). ORF1a encodes 11 NSPs, while ORF1b encodes 5 NSPs. Mapping of the potential starting and stop codons of the NSPs is detailed in (Figure 10B). In the remaining one-third of the genome at the 3′ end, genes encode four primary structural proteins: spike (S), envelope (E), membrane (M), and nucleocapsid (N). This part also contains some NSPs, including NSP3a, NSP3b, NSP3c, NSP7a, and NSP7b. The spike glycoprotein genes consist of 4359 nucleotides in length. The S protein is divided into two subunits (S1 and S2) (Figure 10C). The S1 subunit contains the Receptor Binding Domain (RBD). It is further divided into the N-terminal domain (NTD) and C-terminal domain (CTD). The S2 subunit, or Fusion Domain, contains Fusion Peptide (FP), Heptad Repeat (HR) 1, and the HR2 (Figure 10B). The spike has a potential cleavage site. The S1/S2 Furin cleavage site is mapped at the center of the S gene, which divides the spike into S1 and S2 units. The second cleavage site is at the linker region of the S2, which functions in the binding or host receptors during infection (Figure 10B).

### 3.9. Mapping the Single Nucleotide Polymorphisms (SNIPS) and the Variants Across the Complete Genome Sequences of the New FIPV Field Isolates (A15 and A37)

The multiple sequence alignment based on the complete genome sequencing of the FIPV isolates A15, A37, and FIPV reference isolate (NC_002306) showed 99.9% similarity. Further genome sequence mining analysis identified 22 nucleotide variations between FIPV-A15, FIPV-A37, and FIPV reference isolate 79-1146 (Table 1). Out of these SNIPS, six nucleotide changes were observed in the ORF1a and eight variations in the ORF1b gene. Three nucleotide variations were reported in the spike gene. One nucleotide variation was found in each NSP 3c, E gene, and in the 3′UTR. We observed 34 nucleotide deletions in the NSP 7b gene of the FIPV-A37 isolate, while only one variation was observed in the A15 isolate compared to the FIPV 79-1146 reference isolate (Table 1).

### 3.10. Phylogenetic Analysis of the FIPV Field Isolates (A15 and A37) Based on the Complete Genome Sequences

**Figure 11:**
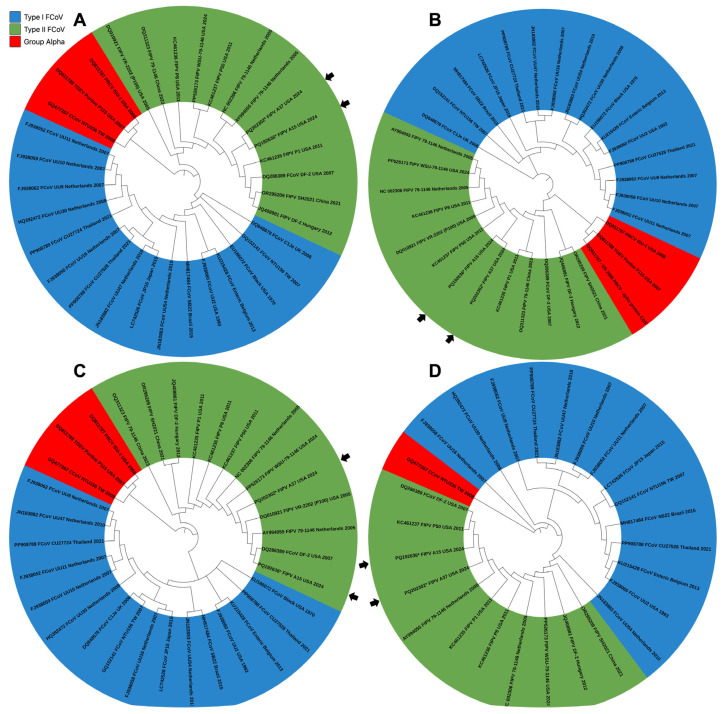
The phylogenetic analysis using the decoded full-length genome, spike, nucleocapsid, and the NSP-7b genes sequences of the FIPV-A15 and FIPV-A37 field isolates. (**A**) The complete genome phylogenetic tree showed the highest Log Likelihood of -218,039.81. (**B**) The tree based on the spike gene sequences showed the highest Log Likelihood of -43,443.84. (**C**) The tree based on the nucleocapsid gene sequences showed the highest Log Likelihood of -8689.05. (**D**) The tree based on the NSP-7b gene sequences showed the highest Log Likelihood of -3224.63. The full-length genomes of FIPV-A15 and FIPV-A37 isolates detected in this study are identified with asterisks (*) and black arrowheads. The phylogenetic trees were generated using the maximum likelihood method and the Tamura–Nei model with bootstrap values (1000 replicates) using MEGA 11 software. The tree was generated using the online software iTOL (version 6). The percentage of the tree in which the associated taxa clustered together is shown next to the branches. The genotype distribution of the isolates is shown on the right side of the Figure.

The phylogenetic analysis was conducted using 32 full-length genomes from FCoV and FIPV isolates, along with isolates from canine coronavirus (CCoV), porcine respiratory coronavirus (PRCV), and transmissible gastroenteritis virus (TGEV). The phylogenetic tree was divided into two groups based on the FCoV serotypes. Type-I FCoV includes isolates from The Netherlands, USA, UK, Thailand, Japan, Brazil, and Belgium collected between 1970 and 2021 (Figure 11A). Type-II FCoV comprised FIPV isolates from the Netherlands, USA, Hungary, and China collected between 2005 and 2024 (Figure 11A). Notably, the FIPV-A15 and FIPV-A37 isolates reported in this study clustered with the sequences of the type-II FCoV group (Figure 11A). Other Alphacoronaviruses, including the CCoV from China (2008), PRCV from the USA (2006), and TGEV from the USA (2007), were positioned between the type-I and type-II FCoV groups in the phylogenetic tree (Figure 11A).

### 3.11. Phylogenetic Analysis of the FIPV Field Isolates (A15 and A37) Using the Sequences of the Spike Glycoprotein, Nucleocapsid, and NSP-7b

To determine the grouping of FCoV isolates based on their spike gene, a phylogenetic tree was constructed using the spike gene sequences of 32 isolates of Alphacoronaviruses (Supplementary Table S1, Figure 11B). The distribution of type-I and type-II FCoV groups in the phylogenetic tree of spike genes mirrored the pattern observed in the full-length genome phylogeny. The FIPV-A15 and FIPV-A37 sequences were consistently clustered with the FIPV group (Figure 11B).

This FIPV/N sequence-based tree was constructed based on sequences of the N gene of some members of the genus alphaviruses (Supplementary Table S1, Figure 11C). The N-based tree analysis of both the FIPV-A15 and the FIPV-A37 showed these isolates were clustered with the N protein sequences of the type-II FIPV serotype (Figure 11C). The CCoV, PRCV, and TGEV are clustered into the alpha coronaviruses (Figure 11C).

The phylogenetic tree was reported using the nucleotide sequences of the FIPV-NSP-7b of some members of the alphaviruses (26 sequences). The tree was divided into two groups based on the FCoV serotypes. The type-I FCoV includes the FCoV from The Netherlands, USA, Taiwan, Thailand, Japan, Brazil, and Belgium, isolated between 1993 and 2021 (Figure 11D). The CCoV isolate NTU3336 from Taiwan (2008) was used as an out-group (Figure 11D). The type-II FCoV includes the FIPV isolates from The Netherlands, USA, Hungary, and China deposited in the GenBank between 2005 and 2024 (Figure 11D). The FIPV-A15 and FIPV-A37 isolates clustered with the sequences of the type-II FCoV group (Figure 11D).

### 3.12. Results of the Pairwise Homology of Novel FIPV Isolates with Coronaviruses from the Alpha Group

**Figure 12:**
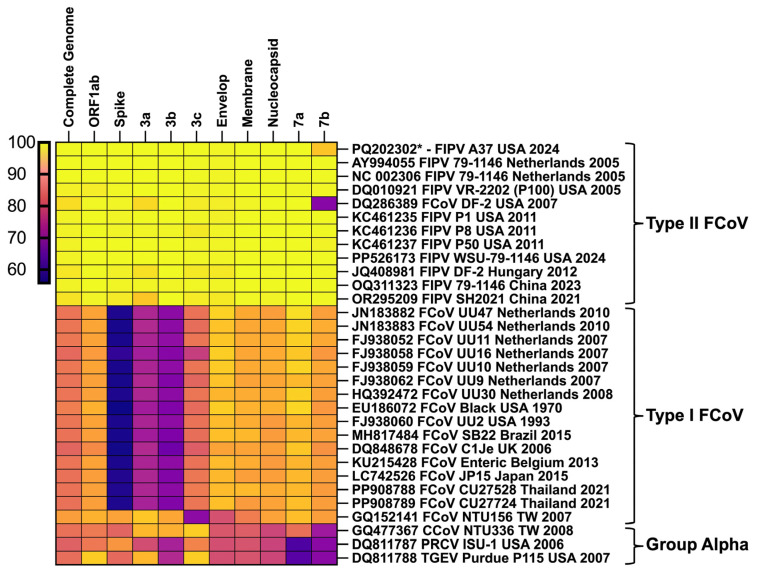
The pairwise homology analysis of FIPV-A15 and FIPV-A37 with alpha coronaviruses. Comparative pairwise analysis was performed for the complete genome, ORF1ab, spike, 3a, 3b, 3c, envelope, membrane, nucleocapsid, 7a, and 7b between 32 alpha coronaviruses isolates. The dataset includes 13 isolates from the type-II FCoV group, 16 isolates from the type-I FCoV group, as well as reference strains for CCoV, PRCV, and TGEV. The FIPV-A15 was used as a standard for the pairwise comparison. The pairwise homology was calculated using Geneious Prime v11. The heatmap was generated using GraphPad Prism v9 software based on the nucleotide similarity level with a color gradient, as indicated in the figure legend.

Comparative pairwise nucleotide sequence alignment of the FIPV-A15 was performed against FIPV-A37 (from this study) reference strains from type-II and type-I FCoV groups, CCoV, PRCV, and TGEV. The FIPV-A15 complete genome exhibited 99.84% similarity with FIPV-A37. Therefore, the FIPV-A15 isolate was used as a reference for further comparisons. The complete genome of FIPV-A15 showed high similarity (97.1–99.9%) with type-II FCoV reference isolates but significantly lower similarity (82.5–85.8%) with type-I FCoV isolates (Figure 12). When compared with other coronaviruses, the complete genome of FIPV-A15 isolates shared 84.13% nucleotide identity with CCoV, 81.73% with PRCV, and 83.69% with TGEV (Figure 12). The spike gene analysis revealed that FIPV-A15 shared over 99% similarity with isolates from the type-II FCoV group, whereas it demonstrated a lower identity range of 56–58% with isolates from the type-I FCoV group. The spike gene of FIPV-A15 also displayed 82.47% similarity with CCoV, 88.95% with PRCV, and 83.16% with TGEV (Figure 12).

### 3.13. Identification of Some Notable Deletions and Mutations in the FIPV-A37 and FIPV-A37 NSP-7b Protein Gene Sequences

**Figure 13:**
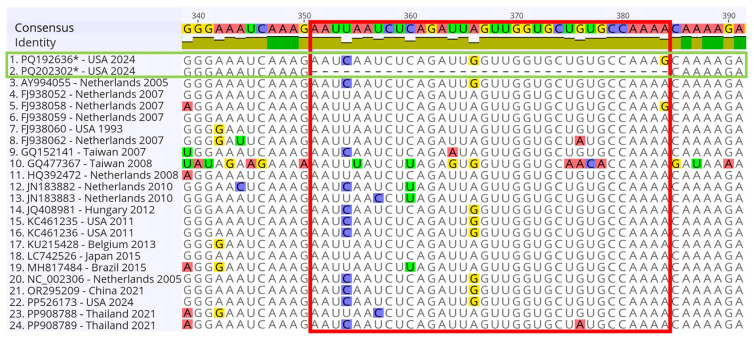
Mapping of some notable deletions and mutations in the FIPV-A37-7b field isolate. The MSA of NSP-7b gene from 24 isolates. The consensus identity at the top represents conserved nucleotide positions across the isolates, with positions labeled based on the NSP-7b gene. Variations are highlighted with distinct colors. The green box indicates the FIPV-A15 and FIPV-A37 isolates generated in the current study. The red box highlights the 34-nucleotide deletion region reported in the FIPV-A37 isolate. The Geneious Prime 2024 Version 11 software was used to generate the MSA.

The multiple sequence alignment using the NSP-7b sequences across the 24 isolates, including FCoV, FIPV, and CCoV, revealed a 34-nucleotide deletion in the FIPV-A37. No other deletions were identified among the aligned isolates within the NSP-7b region (position 351–384) (Figure 13). A unique substitution of adenine (A) to guanine (G) was observed in the FIPV-A15 isolate at position 384 (Figure 13). Interestingly, some mutations were detected within this region in the CCoV (GQ477367), distinguishing it from other FCoV and FIPV isolates.

## 4. Discussion

The multiple sequence alignment was conducted using the (PRCV ISU-1, TGEV-Purdue P115, CCoV NTU336/F/2008, FECoV, and 28 other FIPV and FCoV isolates), and included the A15 and A37 isolates reported in this study. Based on the phylogenetic analysis, these newly filed isolates were clustered with the FECoV type-I and type-II, respectively. The full-length genome sequences of the new FIPV-A15 isolated from a young cat with a severe but localized manifestation of FIPV infection clustered with the type-I FCoV. The FIPV-37, isolated from a middle-aged cat with multisystemic involvement, clustered with the type-II FCoV group.

The non-structural protein (NSP) 2 has hydrophobic residues that are predicted to help in binding the viral replication complex in the Golgi apparatus and inhibit the phosphorylation of eIF-2alpha, thereby inhibiting the host protein synthesis [26]. Therefore, any alteration in the NSP2 sequences may influence the pathogenicity of the FIPV isolates. Previous studies showed that the FIPV WSU 79-1146 isolate (passages 1, 8, and 50) showed minimal nucleotide variations across the viral genome sequences, but the NSP2 protein sequences showed three changes that are continuously showing up across these subsequent passages of the virus in cell culture [14]. In the current study, we reported two nucleotide mutations at positions 796 and 1918 in the NSP2 region of the FIPV-A15 and only one mutation at position 796 in the FIPV-A37 compared to the reference strain (FIPV 79-1146).

The marked alteration in virulence and tissue tropism between FECoV and FIPV is related to some acquired mutations within the C-terminal domains of the FIPV-spike glycoproteins [11]. Three distinct forms of FIPV infections were reported in the affected cats. The wet, the effusive (more prevalent), and the mixed forms [27]. The most notable difference between the FECoV and FIPV on the genomic level is the frequency of mutations across the spike glycoprotein and the structural proteins (3a–c and 7a/b). Some studies reported frequent mutations in the 3C protein of the FIPV genome compared to that of the FECoV [28,29]. Other studies suggested some potential roles of the 3C protein in enteric tissue tropism but which are not essential for the systemic spread of FECoV and FIPV infections [30]. Moreover, mutations that led to the frameshift in the 3C protein may impact the viral tissue tropism and virulence [30]. It has been reported that the 3C protein of the FCoV is a membrane-spanning protein that shares a similar topology with the SARS-CoV-3A protein [31]. A previous study showed that the mutation at F35L of FIPV 79-1146 of the NSP-3C was consistent in several passages, which resulted in the truncation of the NSP-3C protein with 165 aa short [14]. Our results showing the NSP-3C of the FIPV-A15 and the FIPV-A37 showed similarity with FIPV 79-1146, except for one nucleotide substitution of C to T at position 25795 (Table 1).

The NSP-7a and NSP-7b are expressed and considered specific to the members of the Alphacoronavirus, while they are absent in Betacoronaviruses and Gammacoronavirus [32]. One recent study correlated some mutations in the 7b protein with the development of FIPV in FECoV-infected Persian cats [33]. Some studies correlated deletions in the 7b gene with the FIPV adaptability to the cell culture and with the loss of virulence in vitro; however, other studies contradict this notion regarding the impacts of 7b mutations on the FCoV [34]. A study reported truncation of the 7b protein in the FIPV WSU 79-1146 at tissue culture passage 50 and the subsequent passages [14]. Another study reported some reduction in the virulence of the FIPV-WSU 79-1146 at passage 100 and correlated this reduction with the adaptation to cell culture at higher passages [35]. We identified only one substitution, A384G, in the FIPV-A15 7b compared to the FIPV 79-1146. Notably, FIPV-A37 sequences exhibited 34 nucleotide deletions spanning the region (351–384) in the 7b gene, resulting in a truncated protein (Figure 13). A previous study found an absence of the NSP 7 of the TGEV, which shares 72% homology with FIPV-7a, enhanced apoptosis, and led to the translational shutdown [36]. These results suggest that FIPV-37 may be an older isolate of 79-1146, which could explain the differential virulence pattern of the FIPV-A37 isolate compared to the FIPV-A15 and the FIPV 79-1146 reference strain, as observed in this study.

Interestingly, two notable mutations within the spike glycoprotein (M1058L and S1060A) were used to distinguish the FIPV from the FECoV infection in cats [37]. The same study suggests the potential involvement of these mutations in fine-tuning the FIPV tropism and its adaptability to the monocytes and macrophages to enhance viral virulence and pathology [37]. The potential role of the M1058L mutation as a genetic marker for systemic FCoV infection in cats has been proposed [38]. Another study concluded that the M1058L mutation in the spike glycoprotein sequences is a genetic marker that could distinguish between FCoV and FIPV infection in cats [30]. In contrast, the multiple sequence alignment of the S gene of the FIPV 79-1146 of the A15 and A37 sequences showed the presence of aspartic acid (D) at position 1058 instead of methionine (M) or leucine (L). Furthermore, we identified two substitutions in the S gene of the FIPV-A15 compared to the FIPV 79-1146. The first substitution, T2535C, is a synonymous mutation. The second substitution, T3998C, was observed in the S gene of both the FIPV-A15 and the FIPV-A37, resulting in a phenylalanine (F) to serine (S) change at position 1333 (F1333S). Furthermore, a G1923T substitution in the S gene of the FIPV-A37 changed lysine (K) to asparagine (N) at position 641 (K641N). Most coronaviruses continue to alter their genetic materials to evade the host immune response and counteract the effects of antiviral therapies or vaccines.

There are several biological significances of the ongoing mutations across the FIPV genome sequences. The impacts of these mutations mainly depend on the type of these mutations and the target protein. Mutations across the FIPV-S glycoprotein, particularly within the receptor binding domains, will impact the ability of the virus to bind to the target host receptors, such as the aminopeptidase N (APN) [35]. Meanwhile, alteration in the FIPV-S protein sequences may also contribute to the immune evasion of the affected host, which may affect the tissue tropism and virulence of the newly mutated viruses [39]. Furthermore, alteration in the FIPV-NSP3 may also contribute to the viral immune evasion and alteration in the tissue tropism and immune response of the affected cats [13]. On the other hand, mutations in some key proteins, such as the RNA-dependent RNA polymerase (RdRp), may contribute to the viral resistance to some new antiviral therapies, such as the remdesivir or GS-441524 compound [13].

Further studies are required to understand the molecular evolution of the new FIPV isolates. Monitoring the FIPV at the genomic level is highly encouraged to monitor the emergence of any virulent strains that may result in new clinical and pathologic manifestations of the disease. This continuous molecular surveillance system will also help to update the currently used diagnostic assays and develop novel antiviral therapies and vaccines against FIPV infection in cats.

## 5. Conclusions

We reported the full-length genome sequences of two newly filed isolates of the FIPV from feral shelter cats in Long Island, NY. We established the genome structure and organization of these two isolates. The phylogenetic analysis based on the full-length genome sequencing of those two isolates and the sequences of the S and NSP-7b confirm the classification of these two isolates to be clustered with the group II-FCoV along with the other FIPV isolates. Our results confirm the dynamic changes in the FIPV on the genomic level; thus, continuous vigilant monitoring of the virus on the genomic level is highly encouraged.

## Figures and Tables

**Table 1:** Comparison of FIPV-A15 and FIPV-A37 FIPV reference strain 79-1146.

Gene	Position	FIPV 79-1146	A15	A37	Gene	Position	FIPV 79-1146	A15	A37
**ORF1a**	796	G	T	T	ORF1b	17709	G	A	A
	1918	T	C	T		18723	A	G	A
	6406	G	G	A		20352	C	T	C
	7449	T	T	G	Spike gene	22358	G	G	T
	7454	G	G	1bp deletion		22970	T	C	T
	8569	C	T	C		24433	T	C	C
	9543	A	A	C	NSP 3c	25795	C	T	T
**ORF1b**	13135	G	G	T	E gene	26200	T	C	T
	13221	T	A	A	NSP 7b	28785	AAUCAAUCUCAGAUUGGUUGGUGCUGUGCCAAAA	AAUCAAUCUCAGAUUGGUUGGUGCUGUGCCAAAA	34 bp deletion
	13676	C	T	C					
	15316	A	G	G		28818	A	G	
	16347	T	C	C	3′UTR	29137	C	T	C

## Data Availability

All reported sequences out of this study were deposited in the GenBank (PQ192636 and PQ202302). All data will be available upon request from the corresponding author.

## Supplementary Materials

The following supporting information can be downloaded at: https://www.mdpi.com/article/10.3390/v17020209/s1, Table S1: The GenBank accession numbers and the associated information for members of the Alphacoronaviruses that were used in the MSA.
